# Prevalence, New Incidence, Course, and Risk Factors of PTSD, Depression, Anxiety, and Panic Disorder during the Covid-19 Pandemic in 11 Countries

**DOI:** 10.3390/healthcare9060664

**Published:** 2021-06-03

**Authors:** Irina Georgieva, Peter Lepping, Vasil Bozev, Jakub Lickiewicz, Jaroslav Pekara, Sofia Wikman, Marina Loseviča, Bevinahalli Nanjegowda Raveesh, Adriana Mihai, Tella Lantta

**Affiliations:** 1 Department of Cognitive Science and Psychology, New Bulgarian University, 1618 Sofia, Bulgaria; 2 Centre for Mental Health and Society, Bangor University, Bangor LL57 2DG, UK; peter.lepping@wales.nhs.uk; 3 Mysore Medical College and Research Institute, Karnataka 570001, India; 4 Department of Statistics and Econometrics, University of National and World Economy, 1700 Sofia, Bulgaria; v_bozev@unwe.bg; 5 Faculty of Health Sciences, Jagiellonian University Medical College Kraków, 31-008 Kraków, Poland; jakub.lickiewicz@uj.edu.pl; 6 Paramedic Department, Medical College in Prague, 121 08 Prague, Czech Republic; pekarjar@gmail.com; 7 Department of Criminology, University of Gävle, 80176 Gävle, Sweden; Sofia.Wikman@hig.se; 8 Faculty of Medicine, University of Latvia, LV-1004 Riga, Latvia; marina.losevica@lu.lv; 9 Department of Psychiatry, Government Medical College, Mysore 570001, India; raveesh6@yahoo.com; 10 Clinical Department of Medicine, GE Palade University of Medicine, Pharmacy Science and Technology, 540142 Târgu Mureș, Romania; dradrianamihai@yahoo.com; 11 Department of Nursing Science, University of Turku, 20500 Turku, Finland; tella.lantta@utu.fi

**Keywords:** mental health, risk factors, Covid-19, pandemic, resilience, PTSD, anxiety, depression, panic disorder, general population, multinational study

## Abstract

We aimed to evaluate the prevalence and incidence of post-traumatic stress disorder (PTSD), depression, anxiety, and panic disorder (PD) among citizens in 11 countries during the Covid-19 pandemic. We explored risks and protective factors most associated with the development of these mental health disorders and their course at 68 days follow up. We acquired 9543 unique responses via an online survey that was disseminated in UK, Belgium, Netherlands, Bulgaria, Czech Republic, Finland, India, Latvia, Poland, Romania, and Sweden. The prevalence and new incidence during the pandemic for at least one disorder was 48.6% and 17.6%, with the new incidence of PTSD, anxiety, depression, and panic disorder being 11.4%, 8.4%, 9.3%, and 3%, respectively. Higher resilience was associated with lower mental health burden for all disorders. Ten to thirteen associated factors explained 79% of the variance in PTSD, 80% in anxiety, 78% in depression, and 89% in PD. To reduce the mental health burden, governments should refrain from implementing many highly restrictive and lasting containment measures. Public health campaigns should focus their effort on alleviating stress and fear, promoting resilience, building public trust in government and medical care, and persuading the population of the measures’ effectiveness. Psychosocial services and resources should be allocated to facilitate individual and community-level recovery from the pandemic.

## 1. Introduction

The United Nations has described the coronavirus disease (Covid-19) pandemic as humanity’s worst crisis since World War II [1]. Since its emergence in Asia in 2019, the virus has spread to every continent except Antarctica. According to the International Federation of Red Cross and Red Crescent Societies, pandemics are classified as a natural disaster [2]. Natural disasters often have short- and long-term psychological impacts that far exceed the degree of medical morbidity and mortality that ensues [3]. As such, the Covid-19 pandemic is likely to have traumatizing effects on at least parts of the population. Assessing the psychological impacts of the Covid-19 crisis and associated factors is fundamental to inform and tailor the responses of governments and their partner organizations to recover from the crisis.

Therefore, we explored the prevalence, incidence, and course of post-traumatic stress disorder (PTSD), anxiety, depression, and panic disorder by citizens aged 18 and older in 11 countries during the Covid-19 pandemic. We focused on these disorders because studies carried out after natural disasters report a PTSD prevalence ranging from approximately 5% to 60% [4]. There is also evidence that PTSD often co-occurs with panic disorder (PD) [5], while depression and anxiety are the most common mental illnesses worldwide with prevalence ranging from 2% to 6% and 2.5% to 7%, respectively [6]. In addition, given that some etiological and maintenance factors associated with panic disorder (fear conditioning to abnormal breathing patterns, hypervigilance towards breathing abnormalities) overlap with symptoms of Covid-19, one could expect an increased risk of panic disorder following the Covid-19 pandemic [7].

Numerous multinational studies have already explored the prevalence of anxiety and depression during the pandemic and found a pooled prevalence of anxiety and depression varying widely from 11.6% to 58.9% and 16.1% and 69%, respectively [8,9,10,11,12,13,14,15,16,17,18,19,20]. However, none of these multinational studies had reported the prevalence and incidence of PTSD and panic disorder. Findings from a Bangladeshi study yielded high prevalence rates of panic disorder (79.6%) in the general population [21], while a recent systematic review found a PTSD prevalence rate of 21.9% [22]. Furthermore, only one multinational study distinguished between preexisting and emerging mental health disorders as reported by study participants, finding a 14% increase in anxiety symptoms during the pandemic [18]. Therefore, we explicitly assessed the incidence of PTSD, depression, anxiety, and panic disorder related to the pandemic, excluding respondents who reported to have preexisting symptoms of any mental illness before the pandemic or experienced a significant stressful life event during the pandemic that was not directly related to the coronavirus outbreak. In addition, none of these multinational studies have investigated the course of mental disorders by conducting a follow-up assessment. Therefore, we explored if there is a significant improvement or deterioration in respondents’ psychological state 68 days after the first assessment using the same validated instruments to assess their mental state.

Lechat [23] defined a natural disaster, as a “disruption exceeding the adjustment capacity of the affected community”. People usually have the capacity to adjust psychologically after stressful and potentially traumatic events by balancing conflicting needs. However, under the current pandemic, citizens’ needs have been persistently challenged by the containment measures implemented by their national governments to stop the spread of the Covid-19 virus, and citizens were exposed to stress for longer than a year. Most people had to give up favorite hobbies such as travelling, sport, attending restaurants, cafes, cultural and sport events or other entertaining venues, socializing and bonding with friends and loved ones, and even postpone weddings or birthday celebrations. Some people lost their job, their income was reduced, or they or family members were infected by the disease. Simultaneously, people must perpetually adjust to new rules and restrictive policies, many of which seriously curtailed normal civic freedoms [24]. Inevitably, such a complex long-lasting emergency will exhaust many people’ psychological resources to adapt and they will not be able to adjust to the many obstacles associated with the current pandemic. There is also clear evidence that social distancing is associated with a range of poor physical and mental health outcomes [25]. Therefore, this study aimed to explore the impact that the containment measures had on citizens’ mental health. For this purpose, we used concepts like the study participants’ perceived effectiveness, restrictiveness, and compliance with the containment measures; the number of measures that affected each study participant personally; and the number of days that they have been exposed to these measures. In addition to these five factors, we explored the effect of another twenty factors on the psychological wellbeing of citizens. To our knowledge, this is the most comprehensive multinational study exploring the link between four mental disorders and twenty-five associated factors. In addition, we explored the effect of resilience on the development of these mental disorders, because a previous multinational study found that an increase of 1 SD on a resilience score was associated with reduced rates of anxiety and depression across healthcare and non-healthcare professionals [10].

In summary, this study aimed to evaluate the prevalence and incidence of post-traumatic stress disorder, depression, anxiety, and panic disorder, and which risk and protective factors (e.g., resilience) are associated with these mental disorders among citizens in 11 countries during the Coronavirus Disease (Covid-19) pandemic. Furthermore, we explored which factors contribute most to the development of these mental health disorders and evaluated the course of the disorders 68 days after the first assessment.

## 2. Materials and Methods

### 2.1. Study Design and Participants

An online survey was developed via the online platform Typeform for the use in 11 countries, which was translated by the authors into 9 relevant languages. Official translations of the validated instruments were used where applicable. Initially, the authors stipulated which containment measures to stop the spread of Covid-19 were implemented in the country they represent. Only measures that have been applied in at least two countries were included in the survey, resulting in 44 different containment measures. Data were collected via Facebook advertising and the authors’ personal and research networks. To reach more people and to overcome the limited demographic representation on Facebook, we created a website for the project: www.impact-covid19.com (accessed on 1 June 2021). The New Bulgarian University ran paid Facebook marketing campaigns with a budget of €330 (405 USD) per country, targeting all citizens aged 18 and older in the United Kingdom UK, Belgium BE (Flemish region only), the Netherlands NL, Bulgaria BG, the Czech Republic CZ, Finland FI, India IN, Latvia LV, Poland PL, Romania RO, and Sweden SW. The first assessment took place between 26 June and 31 August 2020. The average survey completion time was 24 min. Each participant had to agree and give informed consent to be able to complete the survey. No identifying details were collected, except that some participants agreed to participate in the follow-up assessment and provided a personal email for contact. They were approached two months later, between 26 August and 18 November 2020. The average survey completion time was 12 min. The study coordinator obtained ethical approval from the Research Ethics Committee at the Department of Cognitive Science and Psychology, New Bulgarian University, number Ref №: 279/20 May 2020. Additional ethical approvals were obtained in Poland from the Bioethical Committee of Jagiellonian University Medical College (Ref №: 1072.6120.225.2020), in Latvia from the Academic Ethics Commission of the University of Latvia (on 30 May 2020), in Romania from the Ethics Commission for Scientific Research at the “George Emil Palade” University of Medicine, Pharmacy, Science and Technology in Târgu Mureş (Ref №: 928/26 May 2020), and in India from the Institutional Ethics Committee Mysore Medical College & Research Institute and Associated Hospitals, Mysuru (Ref №: 36120/23 May 2020). For the other countries, the study coordinator’s ethical approval was sufficient.

### 2.2. Procedure and Measures

The survey assessed the participants’ demographic characteristics, their way of coping with the pandemic, their psychological wellbeing, their self-rated compliance with the measures, and their opinion about the effectiveness and restrictiveness of the 44 measures. We used 4 validated instruments at the first and second assessment to assess post-traumatic stress disorder: The Primary Care PTSD Screen for DSM-5 [26], The Generalized Anxiety Disorder (GAD-2) Scale [27], The Patient Health Questionnaire (PHQ- 2) for Depression [28], and The Brief Panic Disorder Severity Scale-Self Report [29]. The GAD-2 consists of two questions and a cut-off score of ≥3 and has been established for identifying likely Generalized Anxiety Disorder with a sensitivity (i.e., true positive rate) of 0.86 (0.76–0.93) and a specificity (i.e., true negative rate) of 0.83 (0.80–0.85) [27]. The PHQ-2 consists of the first two questions of the PHQ-9 and has been shown to be a valid measure of depressive symptoms [30] with a cut-off score of ≥3 for identifying likely Major Depressive Disorder (sensitivity 0.83; specificity 0.92) [28], although a more recent meta-analysis identified a lower pooled sensitivity of 0.76 (0.68–0.82), and a lower pooled specificity of 0.87 (0.82–0.90) [31]. We used the very brief PDSS-SR version consisting of two items with a cut-off score of ≥3 to assess distress during panic attacks and agoraphobic avoidance, which could detect panic disorder with a sensitivity of 85% and a specificity of 66%. In addition to these scales, we used the 4-item Brief Resilient Coping Scale (BRCS) [32] at follow up to assess resilience coping with the Covid-19 crisis. The possible score range on the BRCS is from 4 (low resilience) to 20 (high resilience) with the following interpretation: score ranging from 4 to 13 for “Low resilient copers”, 14–16 for “Medium resilient copers”, and 17–20 for “High resilient copers”. In addition to the validated instruments, the authors reached a consensus on how to formulate relevant questions investigating the participants’ experience with the pandemic. Survey questions reported in this article are described in Table S2 (Supplementary p. 3).

The main study outcome is a positive screening result for PTSD and/or anxiety, and/or depression, and/or panic disorder with cut-off points of ≥3 for all validated scales. Subsequently, four dichotomous variables were created: “Positive PTSD”, “Positive Anxiety”, “Positive Depression”, and “Positive Panic Disorder”. In addition, two more variables were created for respondents who meet the screening criteria for all disorders (i.e., with “Positive PTSD”, “Positive Anxiety”, “Positive Depression”, and “Positive Panic Disorder”) and for respondents who suffer from at least one of these mental disorders.

To assess the containment measures’ impact on participants’ mental health, we calculated the following for each respondent: the average perceived effectiveness (i.e., ‘Average Effectiveness”), restrictiveness (i.e., “Average Restrictiveness”), and compliance (i.e., “Average Compliance”) for all 42 containment measures, and the number of measures that affected each respondent personally (i.e., “Number Measures”). Only two measures (i.e., Contact tracing assessment of Covid-19 transmission and Mass testing for Covid-19) were excluded from the analyses, because they were evaluated during the follow-up assessment and the subsample was smaller (see Supplementary Table S1 p. 2). Furthermore, not all measures were applied in all countries, so we only asked respondents to evaluate the effectiveness of containment measures that were applied in their country. We asked respondents to assess how restrictive they perceived the measures and judge their own compliance with those measures that have affected them personally.

We also calculated the average exposure time to containment measures in days (i.e., “Exposure Time Measures”), starting from the date when each national government implemented the first public health measure related to the pandemic outbreak (i.e., UK: 23-03-2020, BE: 12-03-2020, NL: 12-03-2020, BG: 13-03-2020, CZ: 13-03-2020, FI: 12-03-2020, IN: 05-03-2012, LV: 15-03-2020, PL: 12-03-2020, RO: 18-03-2020, and SE: 12-03-2020), and ending with the respondents’ survey submission date.

The following independent variables were included in the logistic regressions: demographic characteristics (i.e., gender, age, educational level aggregated in 3 groups (i.e., ‘**Lower**’- Primary/High school, ‘**Professional**’—Professional/College degree and ‘**High education**’—Bachelor/Master/ PhD degree)); country of residence; living alone or with others during the pandemic (Yes/No, ‘**Living Alone**’); staff type (i.e., ‘**Medical Staff**’, ‘**Other Essential Staff**’, ‘**Non-essential Staff**’); lost job or income during the pandemic and received financial compensation (Yes/No, ‘**Lost Job with Compensation**’); experienced Covid-19 symptoms and/or being tested positive (‘**Covid-19 Infected**’); family member infected with Covid-19 (Yes/No, ‘**Family Infected**’); concerned about infected family member(s) (from 0 (Not at all concerned) to 10 (Very concerned), ‘**Concerned Family**’); trust in national medical care (from 0 (Strongly distrust) to 10 (Strongly trust), ‘**Trust Medical Care**’); experience of major stressful life event during the pandemic (Yes/No, ‘**With Major Life Event**’); diagnosis or symptoms of mental illness before the pandemic (Yes/No, ‘**With Preexisting Mental Disorder**’); having health conditions (Yes/No, e.g., cardiovascular diseases, diabetes, hepatitis B, chronic obstructive pulmonary disease, chronic kidney disease, liver disease, cancer, morbid obesity, or hypertension; ‘**With Health Conditions**’); experienced stress during the outbreak (from 0 (Not at all stressful) to 10 (Extremely stressful), ‘**Stress Outbreak**’); fear of getting infected with Covid-19 and avoidance (from 1 (No fear or avoidance) to 5 (Extreme, pervasive disabling fear and/or avoidance), ‘**Fear Infection**’); trust in national government (from 0 (Strongly distrust) to 10 (Strongly trust), ‘**Trust Government**’); reaction of national government (from 0 (Not at all sufficient), 5 (Reaction is appropriate) to 10 (Extremely stressful), ‘**Reaction Government**’); truthful response of national government (0 (Very untruthful) to 10 (Very truthful), ‘**Truthful Government**’); hours invested to following the news related to the pandemic (‘**Time News**’); following national containment measures (from 0 (Not at all) to 10 (Strictly every day), ‘**Average Compliance**’); perceived restrictiveness national containment measures (from 0 (Not at all restrictive) to 10 (Extremely restrictive), ‘**Average Restrictiveness**’); perceived effectiveness national containment measures (from 0 (Not at all effective) to 10 (Extremely effective), ‘**Average Effectiveness**’); ‘**Number Measures**’; and ‘**Exposure Time Measures**’.

### 2.3. Statistical Analysis

Data were exported from the survey platform to the statistical program SPSS version 23, which was used to analyze the data. For this analysis we used Descriptive statistics, the Wilcoxon Signed Ranks Test to check the difference between two related samples, Chi-square test, and logistic regression with forward stepwise selection of the factors. Descriptive statistics (mean and proportions) were calculated per country and for the main outcomes. The Wilcoxon’s test was used to compare prevalence and incidence in mental disorders between the first and second assessment. We built four hierarchical stepwise multiple linear regressions with “Positive PTSD”, “Positive anxiety”, “Positive depression”, or “Positive panic disorder” as dependent variables. Dummy variables for the 10 countries were included in the model to control for country differences. A stepwise procedure was used to select the factors with significant predictive value.

## 3. Results

### 3.1. Sample Description

The first assessment was completed by 9942 people from 11 countries. Some people completed the survey multiple times, therefore only the first completion was retained, reducing the number of respondents to 9543 unique responses: UK (*N* = 659), BE (*N* = 384), NL (*N* = 867), BG (*N* = 1862), CZ (*N* = 725), FI (*N* = 543), IN (*N* = 780), LV (*N* = 635), PL (*N* = 996), RO (*N* = 1502), and SE (*N* = 590). Of all respondents, 35% (*N* = 3378) gave permission to be invited via email two months later to participate in the follow-up assessment. On average, this happened 68 days later, when 1926 respondents (57%) completed the second assessment. The demographic characteristics of respondents per country are shown in Table S3 (Supplementary p. 5). Most of the respondents were female (71.4%). To correct for the gender imbalance, the proportion of women was weighted by their real demographic percentage for each country for 2020. The mean age of respondents was 47.5 years (minimum 18, maximum 100), 60% of them had obtained a higher education (bachelor, master, or PhD degree), followed by professional and college education (23.2%) and lower education (16.8%). The majority of the respondents belonged to the group of non-essential staff (76%), followed by other essential staff (12.5%) and medical staff (11.5%). Twenty-six percent of the sample reported that they had lost their job or their income was reduced during the pandemic, but only 17.9% of them have received any financial compensation or aid. Only 8.5% of the respondents reported to have had Covid-19 symptoms and/or have been tested positive, while 21.8% of them reported to have a family member infected with the disease. One-third of the respondents (30.5%) had at least one underlying medical condition exposing them to increased risk of severe Covid-19. Almost one-third of the sample (28.4%) had a history of mental illness, and 36.8% of them experienced a stressful major life event during the pandemic that was not directly related to the pandemic outbreak.

### 3.2. Prevalence and Incidence of PTSD, Anxiety, Depression and Panic Disorder

The prevalence and incidence (presumed diagnosis based on positive screening results) per country is reported in Table S4 (Supplementary p. 6). The average prevalence of PTSD, anxiety, depression and panic disorder for all countries is 32.4%, 28.6%, 30.3%, and 13.7%, respectively. The prevalence of severely ill individuals meeting the screening criteria for all four disorders is 7.3%, while almost half of the study sample suffered from at least one disorder (48.6%). The number of new cases that were associated with the pandemic and met the screening criteria for PTSD, anxiety, depression, and panic disorder are 11.4%, 8.4%, 9.3%, and 3%. A small group of these individuals met the screening criteria for all four disorders (1.5%) and 17.4% developed at least one disorder during the pandemic. There are significant differences across countries in prevalence and incidence of the disorders with Indian respondents reporting the highest prevalence of PTSD (40.8%) and panic disorder (18.8%), with Belgian respondents having the highest prevalence of anxiety (35.2%), and depression being highest in Poland (40.2%). Belgian respondents reported the highest incidence rate of PTSD, anxiety and depression (16.9%, 14.1%, and 14.1%). For panic disorder, the highest incidence rate occurred in Poland (5.8%).

### 3.3. Course of PTSD, Anxiety, Depression, and Panic Disorder

**Figure 1:**
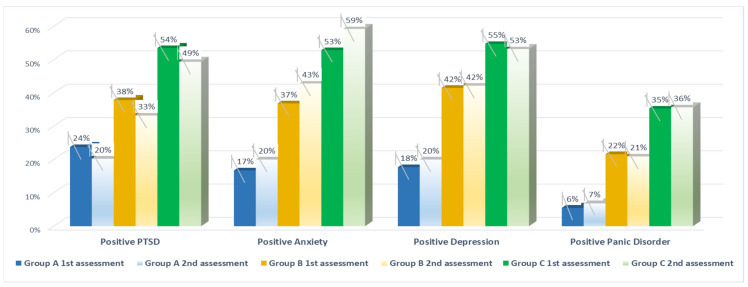
Course of PTSD, anxiety, depression, and panic disorder 68 days after the 1st assessment: Group A: Respondents without preexisting mental illness and no experience of major life event during the pandemic; Group B: With preexisting mental illness and no experience of major life event during the pandemic; Group C: With preexisting mental illness and experience of major stressful life event during the pandemic. “Positive PTSD”, “Positive Anxiety”, “Positive Depression”, and “Positive Panic Disorder” refer to a positive screening result for PTSD, anxiety, depression, and panic disorder respectively, with cut-off points of ≥3 for all validated scales.

Findings about the course of the disorders by comparing three groups (Group A: no pre-existing illness and no additional stressful event; Group B: pre-existing illness but no additional stressful event; Group C: pre-existing illness and additional stressful event) of respondents between the first and second assessment are reported in Table S5 (Supplementary p. 7) and illustrated in Figure 1. The Wilcoxon Signed Ranks Test showed the following significant difference between the groups: PTSD reduced from 24% to 20% (*p* < 0.05), a significant increase in anxiety symptoms between the first and the second assessment for all three groups, respectively, from 17% to 20%, 37% to 43%, and 53% to 59% (*p* < 0.05).

### 3.4. Associated Factors with PTSD, Anxiety, Depression, and Panic Disorder

**Figure 2:**
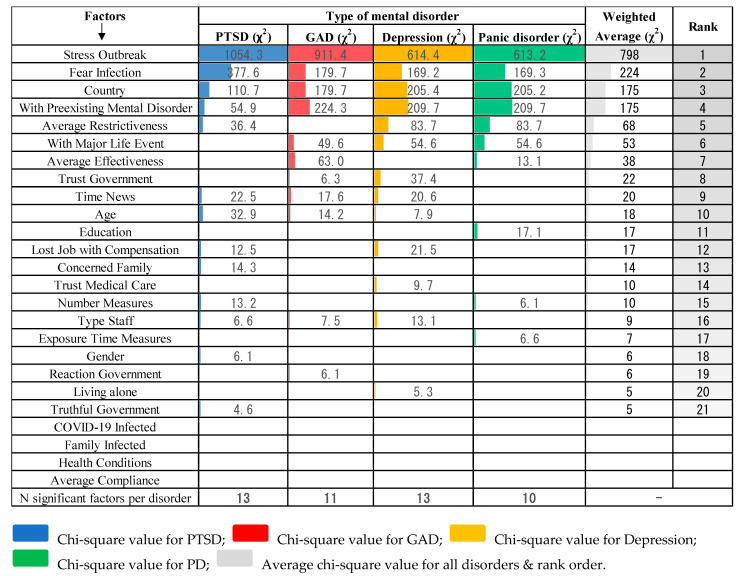
Rank order of associated factors with PTSD, anxiety, depression, and panic disorder according to their Chi-square value (χ^2^).

Findings from the multiple logistic regressions are reported in Table 1. Four factors were excluded by the stepwise selection and did not affect any mental disorder. These were Covid Infected, Family Infected, Health Condition, and Average Compliance. Four other factors affected all four disorders (i.e., Country, With Preexisting Mental Disorder, Stress Outbreak, and Fear Infection), while the remaining factors affected at least one of the disorders. All these factors were ranked according to their largest Chi-square value (χ^2^) obtained as a sum of the individual Chi-square values for each mental disorder, as illustrated in Figure 2.

#### 3.4.1. Factors Associated with PTSD

The first hierarchical linear regression predicting PTSD selected thirteen significant factors: Gender, Age, Country, Staff type, Lost Job with Compensation, Concerned Family, With Preexisting Mental Disorder, Stress Outbreak, Fear Infection, Truthful Government, Time News, Average Restrictiveness, and Number Measures. The stress experienced during the outbreak has the greatest effect on developing PTSD (see Figure 2). Increase in stress by one unit increases the likelihood of meeting the screening criteria for PTSD 1.46 times (CI 95%: 1.40–1.52). The second most important predictor is the fear of getting infected with Covid-19. Each subcategory contributes to a different probability of PTSD, but the highest of them “extreme fear” increases the risk of developing PTSD six times. The third most important factor is the country of residence. All countries are compared to Bulgaria. The results show that the following seven countries have a significantly higher prevalence of PTSD than Bulgaria: United Kingdom, Belgium, Netherlands, the Czech Republic, India, Latvia, and Poland. Among them, the Czech Republic has the highest probability of PTSD—3.6 times (CI 95%: 2.6–4.6) higher than Bulgaria.

Furthermore, we found that having a preexisting mental disorder increases the risk of developing PTSD during a pandemic 1.73 times (CI 95%: 1.47–2.04). The fifth most important predictor is the perceived restrictiveness of the containment measures imposed on citizens by their governments. An increase of the perceived restrictiveness of the measures by one unit increases the risk of suffering from PTSD 1.11 times (CI 95%: 1.07–1.15). Each additional year of age decreases the risk of PTSD 1.02 times (CI 95%: 0.97–0.99). Each additional hour spent on following the news related to the pandemic on TV or social media increases the probability of getting PTSD 1.08 times (CI 95%: 1.04–1.12). Being concerned about family members that they may get infected also increases the risk 1.06 times (CI 95%: 1.03–1.09). Each additional containment measure applied in the country that affects citizens personally increases the likelihood of PTSD development 1.02 times (CI 95%: 1.01–1.03). We also found that people who have not lost their jobs or income were the most resistant toward illness, with the risk of PTSD decreasing 1.70 times (CI 95%: 1.2–2.3). Nonessential staff is exposed to 1.43 times (CI 95%: 1.08–1.89) higher risk of PTSD than medical and other essential staff. It turns out that men are 1.25 times (CI 95%: 0.71–0.95) less likely to develop PTSD than women. When national governments are perceived to be truthful about the pandemic it reduces the risk of PTSD among citizens (1.02 times, CI 95%: 0.96–0.99). All these factors combined predict the incidence of post-traumatic stress disorder (presumed diagnosis based on positive screening results) by 79%.

#### 3.4.2. Factors Associated with Anxiety

The second hierarchical linear regression predicting Generalized Anxiety Disorder (GAD) selected eleven significant factors: Age, Country, Staff type, With Major Life Event, With Preexisting Mental Disorder, Stress Outbreak, Fear Infection, Trust in Government, Reaction of Government, Time News, and Average Effectiveness. The stress experienced during the outbreak is the most important predictor for developing GAD (see Figure 2). Increase in stress by one unit increases the likelihood of meeting the screening criteria for GAD 1.45 times (CI 95%: 1.40–1.50). The second most important predictor is having a preexisting mental disorder, increasing the likelihood of developing GAD 2.87 times (CI 95%: 2.43–3.38). The next most important factor is the country of residence. The results show that the following nine countries have a significantly higher level of GAD than Bulgaria: United Kingdom, Belgium, Netherlands, the Czech Republic, Finland, India, Latvia, Poland, and Sweden. Among them, the Czech Republic has the highest probability for the prevalence of PTSD (4 times higher than Bulgaria). The fourth most important predictor is the fear of getting infected with Covid-19. The highest subcategory “extreme fear” increases ten times the risk of developing GAD.

Furthermore, the fifth most important predictor is the perceived effectiveness of the containment measures imposed on citizens by their governments. Increase in effectiveness by one unit decreases the risk of suffering from GAD 1.08 times (CI 95%: 0.89–0.95). Further, we found that having an experience of a major life event increases the risk of developing GAD during a pandemic 1.71 times (CI 95%: 1.47–1.99). Each additional hour spent on following the news related to the pandemic on TV or social media increases the probability of getting GAD 1.08 times (CI 95%: 1.04–1.12). When increasing age, the risk of GAD decreases 1.02 times (CI 95%: 0.97–0.99) for each additional year. The next factor is the staff group, with “other essential staff” being at greatest risk of developing the disease, 1.6 times (CI 95%: 1.1–2.2) higher than medical staff, which is the group most resistant to GAD. The likelihood of developing anxiety decreases 1.01 times (CI 95%: 0.99–0.99) when trust in the national government is increasing and it increases 1.03 times (CI 95%: 1.00–1.06) with governmental action being perceived as becoming more extreme. All these factors combined predict the incidence of anxiety based on positive screening results by 80%.

#### 3.4.3. Factors Associated with Depression

The third hierarchical linear regression predicting depression selected thirteen significant factors: Age, Country, Staff type, Lost Job with Compensation, Concerned Family, Trust in Medical Care, Living Alone, With Major Life Event, With Preexisting Mental Disorder, Stress Outbreak, Fear Infection, Trust in Government, Time News, and Average Restrictiveness. The stress experienced during the outbreak is the most important predictor for developing depression. Increase in stress by one unit increases the likelihood of meeting the screening criteria for major depressive disorder 1.28 times (CI 95%: 1.24–1.32). The second most important predictor is having a preexisting mental disorder, which increases the risk of depression 2.58 times (CI 95%: 2.21–3.01) (for details, see Figure 2). The next most important factor is the country of residence. The results show that the following nine countries have a significantly higher prevalence of depression than Bulgaria: United Kingdom, Belgium, Netherlands, the Czech Republic, Finland, India, Latvia, Poland, and Sweden. Among them, the Belgium has the highest probability of depression, 3 times higher than Bulgaria. Romania has a significantly lower level of depression compared to Bulgaria with 1.43 times (CI 95%: 0.56–0.89) reduced likelihood. The fourth most important predictor is the fear of getting infected with Covid-19. The highest subcategory “extreme fear” increases the risk of developing depression by as much as 9.6 times (CI 95%: 6.0–15.6).

Furthermore, the fifth most important predictor is the measures’ perceived restrictiveness—an increase in restrictiveness by one unit increases the risk of depression 1.06 times (CI 95%: 1.03–1.06). Furthermore, participants who have experienced a major life event during the pandemic were exposed to 1.67 times (CI 95%: 1.44–1.92) higher risk of developing depression. The likelihood of developing anxiety decreases 1.05 times (CI 95%: 1.02–1.08) when trust in the national government is increasing. We also found that people who have lost their job or income and did not get any financial compensation were exposed to a higher risk of getting depressed 1.43 times (CI 95%: 1.21–1.69). Each additional hour spent on following the news related to the pandemic on TV or social media increases the probability of getting depressed 1.08 times (CI 95%: 1.04–1.12). “Other essential staff” are exposed to a higher risk of getting depressed in comparison with medical staff 1.75 times (CI 95%: 1.26–2.41). Having more trust in the national medical system decreases the risk of getting depressed (1.04 times, CI 95%: 0.93–0.99). In addition, each additional year of age decreases the risk of getting depressed 0.99 times, while people who were under home confinement alone are exposed to 1.2 (CI 95%: 0.62–0.98) higher risk of getting depressed. All these factors combined predict the incidence of depression (presumed diagnosis based on positive screening results) by 78%.

#### 3.4.4. Factors Associated with Panic Disorder

The fourth hierarchical linear regression predicting Panic Disorder (PD) selected the following ten significant factors: Country, Education, With Major Life Event, With Preexisting Mental Disorder, Stress Outbreak, Fear Infection, Average Restrictiveness, Average Effectiveness, Number Measures, and Exposure Time Measures. The most important predictor is stress experienced during the outbreak. Increase in stress by one unit increases the likelihood of having a panic disorder 1.31 times (CI 95%: 1.24–1.38). The second most important predictor is having a preexisting mental disorder, causing an increase of 3.46 times (CI 95%: 2.81–4.26). The results show that the following five countries have a significantly higher prevalence of panic disorder than Bulgaria: United Kingdom, the Czech Republic, India, Latvia, and Poland. Among them, the citizens of the Czech Republic have the highest probability (3 times higher). The fear of infection is the fourth important predictor for developing a PD. The highest subcategory “extreme fear” increases the risk of developing the disorder by a significant 19 times.

Furthermore, the fifth most important predictor is the perceived restrictiveness of the containment measures imposed on citizens by their governments. An increase in restrictiveness by one unit increases the risk of suffering from panic disorder 1.11 times (CI 95%: 1.06–1.16). Further, we found that people who experienced a major life event during the pandemic have a 1.33 times higher risk of developing PD during a pandemic (CI 95%: 1.09–1.63). Having a higher education significantly decreases the risk of developing PD. The next most important predictor is the perceived effectiveness of the containment measures imposed on citizens by their governments. An increase in effectiveness by one unit decreases the risk of suffering from PD 1.07 times (CI 95%: 0.89–0.97). Each additional day living in pandemic conditions increases the risk 1.01 times (CI 95%: 1.00–1.02), while each additional containment measure applied in the country that affects citizens personally increases the likelihood of PD 1.02 times (CI 95%: 1.01–1.03). All these factors combined predict the incidence of Panic Disorder (presumed diagnosis based on positive screening results) by 89%.

### 3.5. Resilience and PTSD, Anxiety, Depression, and Panic Disorder

**Figure 3:**
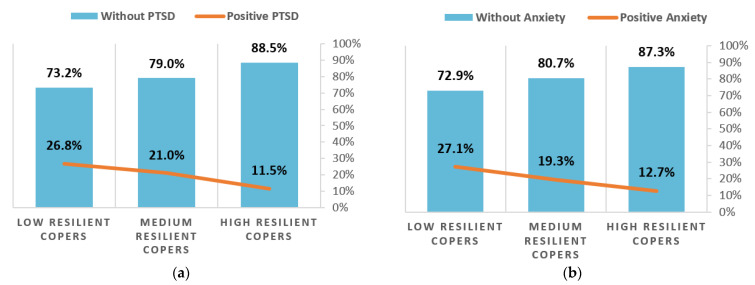

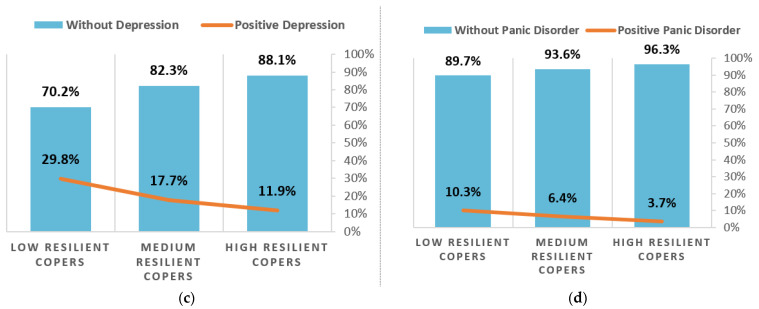
Effect on resilience on mental health: (**a**) Resilience and PTSD. (**b**) Resilience and anxiety. (**c**) Resilience and depression. (**d**) Resilience and panic disorder.

We found a significant relationship between resilience and all disorders using Chi-square test (*p* < 0.05). As illustrated in Figure 3, the same trend can be observed for all disorders: when resilience increases, the percentage of individuals meeting the screening criteria for PTSD, anxiety, depression, and panic disorder significantly decreases.

## 4. Discussion

Our data suggest that 17.4% of participants developed at least one new psychiatric disorder during the pandemic with PTSD being the most common new diagnosis, followed by depression, anxiety, and panic disorder. The average prevalence of PTSD, anxiety, depression, and panic disorder (presumed diagnoses based on positive screening results) for all countries in our study was 32.4%, 28.6%, 30.3%, and 13.7%, respectively. We found that while anxiety increased over the study period, PTSD decreased. The main three factors that most predicted the development of one of the four examined psychiatric illnesses were the amount of stress experienced during the pandemic, the individual fear of getting infected with Covid-19 and having a preexisting mental illness.

Our findings show that medical staff are more resilient against developing PTSD, anxiety, and depression compared with other essential and nonessential staff. These findings are quite unexpected given that other studies found the opposite, showing a similar prevalence of anxiety and depression between healthcare workers and the general public [33], and higher levels of psychological distress among medical staff [34]. However, after conducting additional statistical analyses, we found that the medical staff recruited in our study reported significantly lower burden of preexisting mental disorders before the pandemic in comparison to other essential and nonessential staff. They also experienced significantly less stress from the outbreak and fear of getting infected than nonessential staff. This may explain the discrepancies between our findings and previous studies. Furthermore, medical staff could retain their working habits during the pandemic, were not under home confinement, and were able to socialize at work, all of which may have played an important beneficial in contrast to nonessential workers.

One concerning finding was the number of participants meeting screening criteria for Generalized Anxiety Disorder, which significantly increased over time during the pandemic. This significant increase affected not only participants with preexisting mental illness or those who experienced a significant major life event during the pandemic, but also less vulnerable participants without preexisting mental illness and no experience of major life event. It seems that the longer we are co-existing with the coronavirus under lockdown, the number of people developing anxiety symptoms is exponentially increasing. We found an opposite trend for post-traumatic stress disorder with incidence rate (presumed diagnosis based on positive screening results) significantly decreasing over time. It seems that for most people the PTSD symptoms gradually subside, pointing toward the possibility that at least some of those respondents who screened positive for PTSD may have had an Acute Stress Disorder (ASD) rather than PTSD. ASD is distinguished from PTSD by a symptom pattern that is restricted to a duration of 3 days to 1 month following exposure to the traumatic event.

Governments, policy-makers, and media should not launch public awareness campaigns using fear or threat as a persuasion strategy, although the fear of Covid-19 was previously found to be the most important predictor of public health compliance [35]. This point is emphasized by our finding that the individual fear of infection is the second main predictor of mental health deterioration, increasing the risk of developing PTSD, anxiety, depression, and panic disorder by up to 19%. Some previous studies already emphasized the need for implementing non-fear-based education programs. This emphasizes the importance of education about the effectiveness of particular measures as a decisive tool to persuade citizens to be compliant and reduce the burden of mental disorders during pandemics [12]. This is especially important given our finding that the perceived effectiveness of the measures is a protective factor that significantly reduces the risk of anxiety and panic disorder.

Maintaining a high level of Covid-19 vigilance for safety and containment reasons cannot be criticized. However, in the current situation, where prolonged measures to contain Covid-19 have become inevitable, this study provides clear evidence that a high number and more restrictive containment policies significantly increase the risk of PTSD, depression, and panic disorder in the study participants. In addition, the longer citizens are living in pandemic conditions and must follow containment measures, the greater the risk they are at of developing panic disorder increases. Previous studies found that a complex combination of restrictive containment measures may decrease resilience to Covid-19 and compromise preparations for coexistence with Covid-19 [20]. Although these measures are needed during pandemics to save lives and stop the spread of the virus, governments should refrain from implementing many highly restrictive containment measures for a long period of time whenever possible. They should search for a good balance between the measures’ effectiveness and restrictiveness. A previous study recommended applying the most effective and least restrictive public health measures first [24], and this also seems important for preserving citizens’ mental health. A truthful and transparent governmental policy response significantly decreases the risk of PTSD. Having trust in the national government and medical care are both protective factors, decreasing the risk of developing anxiety or depression. Even under home confinement, people should be encouraged to stay connected and maintain relationships by telephone or video, get enough sleep, eat healthy food, and exercise [36], especially those who live alone; otherwise, they are exposed to a higher risk of developing depression.

People who experienced a major life event during the pandemic that is not directly related to the coronavirus outbreak and people with preexisting mental illness before the pandemic belong to the most vulnerable group being exposed to a higher risk of meeting the screening criteria of any of the studied disorders. These factors place a high level of responsibility onto existing mental health services to try and actively support people with preexisting mental illness and psychiatric patients under their care. Resources ought to be made available to facilitate this in order to avoid a later higher mental illness burden for the population. To preserve their mental health, people should limit the time following the news related to Covid-19 on TV, radio, newspapers or social media because we found that each additional hour spent increases the probability of getting PTSD, anxiety, or depression. The frequent exposure to social media/news concerning Covid-19 has been found to be a risk factor elsewhere, too [37].

Overall, it is urgent that political and health authorities pay attention to the mental health of infected and uninfected individuals during the pandemic given that almost one fifth (17.4%) of participants develops at least one of the mental disorders investigated in this study. It is necessary to provide additional funding for prevention and treatment strategies, as poorer mental health can be associated with shorter life expectancy [38,39,40,41], high economic burden [42], and higher suicide rates [43,44]. Suicide prevention in the context of Covid-19-related unemployment has been found to be a critical priority [45]. The Latvian Ministry of Health has already allocated €7 million to reduce the negative impact of Covid-19 on public mental health [46]. More governments should follow this example, especially in countries where individual or group psychotherapy is not covered by the national health care system or health insurance schemes (e.g., in Bulgaria). This is very important given the fact that people who suffered job or income loss caused by the pandemic and did not get any financial compensation for it are exposed to a higher risk of depression, and they lack financial resources to cover their treatment. To provide psychosocial services to facilitate individual- and community-level recovery from the pandemic is a key recommendation given by the WHO [47].

Efforts should be made to support individual resilience of the population relating to Covid-19, as we found that high resilience is significantly associated with lower prevalence of PTSD, anxiety, depression, and panic disorder. A recent study [48] investigating the impact of a brief educational video intervention on perceived knowledge, perceived safety, and the individual resilience of the population relating to the Covid-19 outbreak and reported a significant overall increase in all examined variables including resilience and perceived safety. This is also an important outcome in light of our study finding that fear of infection increases the likelihood of developing a mental illness during the pandemic period.

Belgian respondents reported the highest new incidence of anxiety, depression, and PTSD across all countries studied, and the highest prevalence of anxiety (presumed diagnosis based on positive screening results). We did not investigate the effect of loneliness and low social support on mental health, but a study among young Belgium people found these factors to be the main predictors of mental distress [49]. Another study in Belgium found psychological distress to be associated with the consequences of the Covid-19 pandemic and containment measures [50]. Furthermore, Polish respondents reported the highest prevalence of depression and the highest new incidence of panic disorder (presumed diagnosis based on positive screening results). This is probably associated with the finding that Polish respondents had the lowest trust in their national government and medical care and perceived the reaction of their government as most untruthful. The Polish government had applied a hard lockdown at the beginning of the pandemic. As a result, many people were not allowed to leave their houses for many months, some of them lost their jobs, increasing their insecurity and isolation. India had the highest prevalence of PTSD and panic disorder (presumed diagnoses based on positive screening results). Indian respondents reported being personally affected by the highest number of containment measures (*N* = 21), which is in keeping with the fact that the Indian police and special forces strictly enforced the implementation of the Indian Epidemic Act. Maybe such a strict lockdown policy of “drones to monitor physical distancing during lockdown and the application of a cluster containment strategy (if three or more patients are diagnosed, all houses within 3 km are surveyed to detect further cases, trace contacts, and raise awareness) [51]” has taken its toll on India’s citizens’ mental health. Not all differences between countries can be easily explained and more research would be useful in this area.

Several limitations have been noted previously in relation to this study [24]. First, as the survey was web-based and recruitment was largely through social media, we acknowledge the potential for selection bias. We cannot assume that our study population is representative of the older population who do not use social media as frequently as younger people [52]. Second, although the sample size is large and data were collected in eleven countries, Russian, French, and Swedish speaking people were not recruited in Latvia, Belgium, and Finland, respectively. Third, the number of completed survey responses was much higher among Bulgarian residents. This is unsurprising as the New Bulgarian University ran the social media marketing campaigns. We also acknowledge that our results might not fully depict citizens’ experience with the measures as most data were collected in August, when countries were easing the lockdown restrictions after the first wave of the Covid-19 pandemic. In addition, we need to acknowledge that we did not ask study participants if they were under quarantine at the time of the survey. This is important because previous study found that populations in quarantine show significantly higher risks of depression and insomnia, when compared to the general population [53]. While prevalence studies examining common mental illnesses vary widely in different populations, our prevalence numbers (presumed diagnoses based on positive screening results) are generally higher than what we would normally expect in a population study. This may be explained by a genuine increase in prevalence during the pandemic when we started the survey. Alternatively, there could have been an element of self-selection bias in those answering the survey or an overestimate towards false positives.

## 5. Conclusions

The Covid-19 pandemic has a severe impact on citizens’ mental health causing almost one-fifth (17.4%) of the study participants to develop at least one major mental disorder. Stress during the pandemic period and fear of getting infected are major factors that negatively affect people’s mental health. Media and public health campaigns should focus on alleviating stress and fear as well as promoting resilience. In addition, they should maintain and build public trust in public health authorities and medical care and persuade the population of the effectiveness of any public health measure during a pandemic. To preserve their citizens’ mental wellbeing during pandemic emergencies, governments should refrain from implementing many highly restrictive containment measures for a long period of time if possible and should provide a truthful and transparent response to the pandemic. Additional psychosocial services and resources should be allocated to facilitate individual and community-level recovery from the pandemic.

## Figures and Tables

**Table 1:** Output from the multiple linear regressions with “Positive PTSD”, “Positive anxiety”, “Positive depression”, or “Positive panic disorder” as dependent variable and 25 independent factors.

Factor 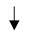	Type of Mental Disorder											
	PTSD Correct Class = 79% N = 5288			GAD Correct Class = 80% N = 5288			Depression Correct Class = 78% N = 5288			Panic Disorder Correct Class = 89% N = 5162		
	Effect	*p*-Value	Exp (B)	Effect	*p*-Value	Exp (B)	Effect	*p*-Value	Exp (B)	Effect	*p*-Value	Exp (B)
**Gender** (Female = 0)	−	0.010	0.82									
**Age**	−	<0.001	0.98	−	<0.001	0.99	−	<0.001	0.99			
**Country** (Bulgaria = 0)		<0.001			0.00			<0.001			<0.001	
United Kingdom	+	0.023	1.49	+	0.002	1.74	+	<0.001	1.96	+	0.031	1.65
Belgium	+	0.001	2.16	+	<0.001	3.18	+	<0.001	3.01			
Netherlands	+	0.008	1.63	+	<0.001	2.09	+	<0.001	1.79			
Czech Republic	+	<0.001	3.58	+	<0.001	3.99	+	<0.001	2.68	+	<0.001	2.97
Finland				+	0.001	1.87	+	0.015	1.94			
India	+	0.002	1.71	+	<0.001	2.51	+	<0.001	1.54	+	0.009	1.78
Latvia	+	<0.001	1.94	+	<0.001	2.26	+	<0.001	2.28	+	<0.001	2.19
Poland	+	0.026	1.36	+	<0.001	3.01	+	<0.001	2.91	+	<0.001	2.68
Romania							−	0.003	0.70			
Sweden				+	<0.001	2.65	+	0.004	1.83			
**Education** (Lower education = 0)											<0.001	
Professional and College education										−	0.014	0.66
High education										−	<0.001	0.54
**Type Staff** (Medical Staff = 0)		0.041			0.027			0.003				
Other essential staff				+	0.011	1.56	+	0.001	1.75			
Nonessential staff	+	0.012	1.43	+	0.013	1.44	+	0.016	1.38			
**Lost Job with Compensation** (No Lost Job = 0)		0.003						<0.001				
Lost job & no payment							+	<0.001	1.43			
Lost job & payment	+	0.002	1.70									
**Covid Infected** (No = 0)												
**Family Infected**												
**Concerned Family**	+	<0.001	1.06									
**Trust Medical Care**							−	0.002	0.96			
**Living alone** (Alone = 0)							−	0.021	0.82			
**With Major Life Event** (No = 0)				+	<0.001	1.71	+	<0.001	1.67	+	0.004	1.33
**With Preexisting Mental Disorder** (No symptoms = 0)	+	<0.001	1.73	+	<0.001	2.87	+	<0.001	2.58	+	<0.001	3.46
**Health Condition** (No conditions = 0)												
**Stress Outbreak**	+	<0.001	1.46	+	<0.001	1.45	+	<0.001	1.28	+	<0.001	1.31
**Fear of Infection** (None = 0)		<0.001			<0.001			<0.001			<0.001	
Mild	+	<0.001	2.22	+	0.002	1.39	+	0.001	1.37	+	<0.001	2.84
Moderate	+	<0.001	3.72	+	<0.001	2.58	+	<0.001	2.27	+	<0.001	10.35
Severe	+	<0.001	6.50	+	<0.001	5.05	+	<0.001	3.66	+	<0.001	13.28
Extreme	+	<0.001	6.00	+	<0.001	9.99	+	<0.001	9.65	+	<0.001	19.10
**Trust Government**				−	0.002	0.99	−	<0.001	0.95			
**Reaction Government**				+	0.015	1.03						
**Truthful Government**	−	0.033	0.98									
**Time News**	+	<0.001	1.08	+	<0.001	1.08	+	<0.001	1.08			
**Average Restrictiveness**	+	<0.001	1.11				+	<0.001	1.06	+	<0.001	1.11
**Average Compliance**												
**Average Effectiveness**				−	<0.001	0.92				−	0.001	0.93
**Number Measures**	+	<0.001	1.02							+	0.014	1.02
**Exposure Time Measures**										+	0.008	1.01

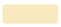
 Not significant factors (*p* ≥ 0.05); 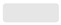 Factors excluded from the analyses due to stepwise selection; “+”factor’s positive effect on the mental disorder; ‘-’ factor’s negative effect on the mental disorder.

## Data Availability

The data presented in this study will be available after data anonymization at https://zenodo.org/ (accessed on 28 May 2021) when all data from this international study are analyzed and reported/published. The data are not currently publicly available due to privacy restrictions.

## Supplementary Materials

The following are available online at https://www.mdpi.com/article/10.3390/healthcare9060664/s1, Table S1: Type and number of containment measures implemented by countries, marked with ‘X’, Table S2: Survey questions reported in this study, Table S3: Demographics & variations across countries (%, mean), Table S4: Prevalence and incidence of PTSD, anxiety, depression, and panic disorder per country, Table S5: Course of PTSD, anxiety, depression, and panic disorder.
