# Supplementary material for: Prevalence, New Incidence, Course, and Risk Factors of PTSD, Depression, Anxiety, and Panic Disorder during the Covid-19 Pandemic in 11 Countries
Source: Healthcare (Basel). 2021 Jun 3;9(6):664. doi: 10.3390/healthcare9060664 (PMC8227861; doi:10.3390/healthcare9060664)
Supplement: Supplementary file 1 [file healthcare-09-00664-s001.zip › healthcare-1193258-supplementary.pdf]

Table S1. Type and number of containment measures implemented by countries, marked with 'X'

Type of containment measure	England	Nether-lands	Finland	Sweden	Bulgaria	Romania	Poland	Czech Republic	Latvia	India
Hygienic and health measures										
1. Recommendation on washing hands more frequently	X	X	X	X	X	X	X	X	X	X
2. Keeping respiratory hygiene by mandatory wearing a mask in public places					X	X	X	X		X
3. Mandatory testing of all suspected cases*							X			
4. Recommendation on coughing or sneezing into your elbow	X	X	X	X	X	X	X		X	
5. Recommendation on NOT shaking hands	X	X	X	X	X	X	X	X	X	X
6. Mandatory reporting symptoms of illness to health authorities		X					X		X	X
7. Enhanced cleaning and disinfection procedures		X	X	X	X	X	X	X	X	
Social-distancing measures										
8. Cancellation of all mass gatherings and events (cultural, sport, scientific or religious)	X	X	X	X	X	X	X	X	X	X
9. Funerals and weddings are forbidden, or the number of attendees is restricted	X	X	X	X	X	X	X	X	X	>20
10. Closure of preschools, nurseries, and primary schools	X	X			X	X	X	X	X	X
11. Closure of secondary schools, colleges and universities	X	X	X	X	X	X	X	X	X	X
12. Final exams are cancelled or postponed	X				X		X		X	X
13. Recommendation on not using public transportation (e.g. bus, trains, ships, etc.)	X	X		X	X	X		X	X	X
14. Recommendation on working from home if possible	X	X	X	X	X	X	X		X	X
15. Keep at least 1-2 meters away from other people	X	X	X	X	X	X	X	X	X	X
16. Mandatory stay-at-home, unless it is absolutely essential to leave the home	X				X	X	X	X		X
17. Introducing market / shopping hours for vulnerable groups	X			X	X	X	X			Given at door steps free of cost
18. A ban on visiting national and nature parks and mountains	X				X	X	X			X
19. Dissolution of the Parliament - National Assembly meets only on bills and acts related to the state of emergency	Virtual meetings				X					X
20. Closure of pubs, cafes, restaurants, except for delivery and takeaway services.	X	X	X		X	X	X	X		X
21. Closure of cinemas, theatres, opera, concert venues, libraries, museums, heritage sites, discos, gambling halls, sport indoor and outdoor facilities.	X	X	X		X	X	X	X	X	X
22. Closure of non-essential shops and large shopping malls.	X				X	X		X		X
23. Limited number of citizens are allowed to gather and visit open and closed public places.	X				X	More than 3	X		X	
24. Closure of playgrounds	X	X			X	X	X	X	X	X
25. Closure of all hotels and tourist accommodation	X				X	X		X		X
26. Closure of the judiciary and courts					X		X		X	Virtual meeting
27. Discontinued are all elective medical surgeries and procedures	X	X		X	X	X	X	X	X	X

Type of containment measure	England	Nether-lands	Finland	Sweden	Bulgaria	Romania	Poland	Czech Republic	Latvia	India
28. Visits to housing services for the elderly and other at-risk groups are prohibited		X	X	X		X			X	
29. A curfew order						X				X
30. People are not allowed to receive guests in their home or the number of guests is restricted		X				X		X	X	X
Isolation of symptomatic individuals and quarantine										
31. Isolation of ill persons at home for certain period of time	X	X	X		X	X	X	X	X	
32. Obligatory admission to a hospital of seriously ill persons					X	X	X	X		
33. Placement in mandatory home quarantine of contacts		X	X		X	X	X		X	X
34. Placement in mandatory home quarantine of citizens returning from abroad			X		X	X	X	X	X	X
Travel restrictions and border controls										
35. Placement of cordon sanitaire on areas infected by a disease such as big cities (i.e. a guarded line preventing anyone from leaving the infected area)					X	X				X
36. Using screening procedures such as asking travelers if they have symptoms of influenza; have had close contact with someone with influenza; performing a visual screen for signs of influenza or measuring body temperature		X	X		X	X	X	X	X	X
37. Border closures: partial or total closure of a land border (e.g. to people from outside the EU)		X	X		X	X	X	X	X	X
38. Flight suspensions: government bans on flights from or to the country		X			X	X	X	X	X	X
39. Present a health screening form when entering the country		X				X		X	X	X
40. Citizens are not allowed to leave the country			X				X			X
Surveillance measures										
41. Contact tracing assessment of Covid-19 transmission		X	X		X		X	X		
42. Police forces are allowed to request and obtain citizens' personal information from internet and telephone providers					X				X	
43. Mass testing for Covid-19 (i.e. expanded targeted testing or random testing of the general population, irrespective of symptoms)		X	X							
Other measures										
44. Declaring state of emergency in the country			X		X	X		X	X	X
45. Penalties or fines for non-compliance with covid-19 containment measures	X	X	X		X	X	X	X	X	X
**Total number of measures: 45**	**24**	**27**	**22**	**13**	**37**	**36**	**33**	**28**	**30**	**35**

*Excluded because it was only used in 1 country

Table S2. Survey questions reported in this study

Question	Answer
1. What is your gender?	A. Female<br>B. Male<br>C. Other
2. In which country do you currently reside?	A. Belgium<br>B. Bulgaria<br>C. Czech Republic<br>D. United Kingdom<br>E. Finland<br>F. India<br>G. Latvia<br>H. Nederland<br>I. Poland<br>J. Romania<br>K. Sweden
3. Which year were you born?	
4. What is the highest level of schooling you have completed or the highest degree you have received?	A. Primary school<br>B. High school<br>C. Professional qualification (not university)<br>D. College degree<br>E. Bachelor's degree<br>F. Master's degree<br>G. Doctoral degree
5. Did you belong to the group of essential staff (e.g. medical staff, food preparation & serving workers, stock clerks and order fillers, etc.) or nonessential workers during the coronavirus outbreak?	A. Medical staff<br>B. Other essential staff<br>C. Nonessential staff
6. Have you lost your job or income due to the containment measures taken by your national government?	A. No, I did not<br>B. I lost my job<br>C. My working hours were reduced<br>D. I had to take unpaid leave<br>E. I was furloughed
7. Have you gotten a COVID-19 Pandemic Unemployment Payment or other types of financial compensation?	A. Yes<br>B. No
8. Have you been infected and tested positive for the coronavirus?	A. No<br>B. I have experienced COVID-19 symptoms, but I have not been tested<br>C. I have experienced COVID-19 symptoms and have been tested positive<br>D. I have been tested positive, but I have not experienced any COVID-19 symptoms
9. Have any of your family members or close friends been infected with the coronavirus?	A. No<br>B. Yes<br>C. Possibly
10. How much are you concerned that family members or close friends may get infected with the virus?	Measured on 11-point Likert scale from 0 (Not at all concerned) to 10 (Strongly concerned)
11. Have you been living alone or with relatives during the coronavirus outbreak?	A. Alone<br>B. With relatives / other people
12. Have you experienced any significant stressful life event lately that is not directly related to the coronavirus outbreak?	A. Yes<br>B. No
13. Have you been diagnosed or experienced any symptoms of depression, anxiety, panic attacks or posttraumatic stress disorder before the coronavirus outbreak?	A. No<br>B. Depression<br>C. Anxiety<br>D. Panic attacks<br>E. Posttraumatic stress disorder<br>F. Other mental illness
14. Are you having any of the following health conditions: cardiovascular diseases, diabetes, hepatitis B, chronic obstructive pulmonary disease, chronic kidney disease, liver disease, cancer, morbid obesity, and hypertension?	A. Yes<br>B. No
15. How many hours per day on average have you been engaged in following the news related to the coronavirus outbreak on TV, radio, newspapers or social media?	

16. How much do you trust that the hospitals in your country have the resources and expertise to provide the best treatment available to people infected with the coronavirus?	Measured on 11-point Likert scale from 0 (Strongly distrust) to 10 (Strongly trust)
17. How much do you trust your national government to take care of its citizens?	Measured on 11-point Likert scale from 0 (Strongly distrust) to 10 (Strongly trust)
18. Do you think the reaction of your national government to the coronavirus outbreak has been appropriate, too extreme or not sufficient?	Measured on 11-point Likert scale from 0 (Not at all sufficient), 5 (Reaction is appropriate) to 10 (Extremely stressful)
19. How factually truthful do you think your national government has been about the coronavirus outbreak?	Measured on 11-point Likert scale from 0 (Very untruthful) to 10 (Very truthful)
20. How stressful have you found the crisis with the coronavirus outbreak?	Measured on 11-point Likert scale from 0 (Not at all stressful) to 10 (Extremely stressful)
21. During the past week were there any social situations (e.g. work, public transportation, movie, theaters, crowds, shopping malls) you avoided, or felt afraid of (uncomfortable in, wanted to avoid or leave), because of fear of getting infected by the coronavirus? Are there any other situations that you would have avoided or been afraid of if they had come up during the past week, for the same reason? Please rate your level of fear and avoidance this past week.	1-None: no fear or avoidance; 2-Mild: occasional fear and/or avoidance but I could usually confront or endure the situation. There was little or no modification of my lifestyle due to this; 3-Moderate: noticeable fear and/or avoidance but still manageable. I avoided some situations, but I could confront them with a companion. There was some modification of my lifestyle because of this, but my overall functioning was not impaired; 4-Severe: extensive avoidance. Substantial modification of my lifestyle was required to accommodate the avoidance making it difficult to manage usual activities; 5-Extreme: pervasive disabling fear and/or avoidance. Extensive modification in my lifestyle was required such that important tasks were not performed.
22. Has this measure been applied in your country?	A. Yes, this measure has been applied in my country and affected me personally -> Go to Question 17 & 18<br>B. Yes, this measure has been applied in my country, but did not affect me personally -> Go to Question 19<br>C. No, this measure has not been applied in my country -> Go to next measure<br>D. I don't know -> Go to next measure
23. To what extent have you followed this measure? Please try to be as honest as possible. Your answers will be kept confidential.	Measured on 11-point Likert scale from 0 (Not at all) to 10 (Strictly every day)
24. To what extent has this measure caused you any discomfort or restricted your personal freedom and your fundamental human rights to engage in work, education, meet other people, move freely within the country or to visit other countries?	Measured on 11-point Likert scale from 0 (Not at all restrictive) to 10 (Extremely restrictive)
25. How effective do you find this measure to prevent the spread of COVID-19 virus?	Measured on 11-point Likert scale from 0 (Not at all effective) to 10 (Extremely effective)
26. Would you like to participate in the follow-up assessments of this study by answering the questions above after a month?	A.Yes<br>B.No

Table S3. Demographics & variations across countries (%, mean)

Characteristics / Variables	UK N=659	Belgium N=384	Netherlands N=867	Bulgaria N=1862	Czech Rep N=725	Finland N=543	India N=780	Latvia N=635	Poland N=996	Romania N=1502	Sweden N=590	Total N=9543
Female (%)	59.2	75.0	68.7	80.2	85.4	82.9	30.9	85.7	79.2	63.2	77.6	71.4
Age (mean)	56.9	52.6	55.1	43.4	40.9	50.2	47.7	45.4	38.1	50.5	51.8	47.5
Lower Education<br>Professional Education<br>Higher Education (BA, MA, PhD) (%)	18.5<br>39.8<br>41.7	24.2<br>22.9<br>52.9	15.0<br>68.7<br>16.3	10.0<br>10.3<br>79.6	45.5<br>20.0<br>34.5	18.2<br>55.1<br>26.7	4.2<br>13.5<br>82.3	8.3<br>20.9<br>70.8	26.0<br>4.8<br>69.2	19.0<br>9.3<br>71.7	2.9<br>34.2<br>62.9	16.8<br>23.2<br>60.0
Medical Staff (%)<br>Other Essential Staff (%)<br>Non-essential Staff (%)	7.9<br>21.9<br>70.2	9.6<br>15.4<br>75.0	12.0<br>18.9<br>69.1	4.3<br>6.3<br>89.4	31.3<br>10.8<br>57.9	14.9<br>10.3<br>74.8	28.6<br>14.4<br>57.0	11.2<br>18.9<br>69.9	4.3<br>11.0<br>84.7	5.2<br>7.5<br>87.3	16.4<br>20.5<br>63.1	11.5<br>12.5<br>76.0
Lost Job (%)	23.8	14.1	16.3	35.1	23.5	13.1	38.6	25.5	35.9	24.1	8.5	26.0
Received Covid-19 Unemployment Compensation of Lost Job (%)	35.0	53.7	29.1	10.1	25.6	36.6	9.0	19.1	16.8	14.6	26.0	17.9
With Covid-19 symptoms and/or tested positive (%)	16.4	10.9	15.9	7.0	8.1	7.9	4.4	4.3	4.7	3.2	23.2	8.5
Family Infected with Covid-19 (%)	32.2	36.5	29.2	16.3	14.3	13.6	25.5	9.6	13.9	18.7	53.9	21.8
Concerned Family (mean)	4.4	4.9	3.1	5.5	4.4	6.4	5.9	5.5	4.4	5.6	6.6	5.1
With Preexisting Mental Disorder (%)	35.5	26.3	18.7	33.1	23.6	41.1	27.4	26.5	21.6	25.9	36.8	28.4
With Major Life Event (%)	30.2	31.0	26.4	31.3	44.1	37.4	42.8	48.5	44.4	38.9	31.5	36.8
With Health Conditions (%)	30.5	27.6	32.5	23.2	27.7	37.8	30.8	32.6	28.3	36.9	34.7	30.5
Time News (mean in hours)	1.8	1.7	2.1	1.9	1.8	1.3	2.1	1.2	1.5	2.2	1.5	1.8
Trust Hospitals (mean): measured on 11-point Likert scale from 0 (Strongly distrust) to 10 (Strongly trust)	5.8	6.7	5.7	3.3	6.6	7.7	5.2	5.3	2.9	3.0	6.7	4.7
Trust Government (mean): measured on 11-point Likert scale from 0 (Strongly distrust) to 10 (Strongly trust)	2.9	3.5	3.0	2.1	3.1	6.6	5.7	3.9	1.3	3.0	5.9	3.3
Reaction Government (mean): measured on 11-point Likert scale from 0 (Not at all sufficient), 5 (Reaction is appropriate) to 10 (Extremely stressful)	5.0	5.9	7.1	5.8	6.2	5.2	5.2	6.8	6.1	5.1	3.9	5.7
Truthful Government (mean): measured on 11-point Likert scale from 0 (Very untruthful) to 10 (Very truthful)	2.6	5.9	6.9	3.2	4.1	6.2	5.5	4.9	1.7	3.7	6.3	4.2
Stress Outbreak (mean): measured on 11-point Likert scale from 0 (Not at all stressful) to 10 (Extremely stressful)	6.2	6.7	5.9	6.8	5.7	6.2	6.4	5.6	6.1	7.7	6.0	6.5
Fear of Infection (mean)	1.9	2.0	1.6	2.0	1.6	2.0	2.3	1.7	1.9	2.0	2.5	1.9
Average Effectiveness (mean)	5.2	5.7	3.8	5.7	5.8	7.7	8.2	5.7	4.8	6.2	7.4	5.9
Average Restrictiveness (mean)	5.9	6.4	7.2	5.8	6.6	4.0	5.5	5.5	6.1	6.8	3.8	5.9
Average Compliance (mean)	7.5	8.1	6.2	8.2	8.1	8.7	8.8	7.9	7.3	8.3	8.6	8.0
Number Measures	14	16	12	18	15	12	21	15	16	13	8	15
Exposure Time Measures (days)	139	154	153	150	145	145	152	155	153	145	148	149

Table S4. Prevalence and incidence of PTSD, anxiety, depression, and panic disorder per country

Country →	UK N=659		Belgium N=384		Netherlands N=867		Bulgaria N=1862		Czech Rep N=725		Finland N=543		India N=780		Latvia N=635		Poland N=996		Romania N=1502		Sweden N=590		Total N=9543	
**Mental disorder**	**Prevalence[1] %**	**Incidence[2] %**	**Prevalence[1] %**	**Incidence [2] %**	**Prevalence [1] %**	**Incidence [2] %**	**Prevalence[1] %**	**Incidence [2] %**	**Prevalence [1] %**	**Incidence [2] %**	**Prevalence [1] %**	**Incidence [2]%**	**Prevalence [1] %**	**Incidence [2] %**	**Prevalence [1] %**	**Incidence [2] %**	**Prevalence [1] %**	**Incidence [2] %**	**Prevalence [1] %**	**Incidence [2] %**	**Prevalence[1] %**	**Incidence[2] %**	**Prevalence[1] %**	**Incidence[2] %**
**Positive PTSD***	27.0	9.1	37.0	16.9	25.1	10.8	33.4	12.9	39.0	13.9	20.6	5.0	40.8	11.4	25.7	7.4	31.4	11.2	38.0	13.1	29.5	9.7	32.4	11.4
**Positive GAD***	28.4	5.8	35.2	14.1	27.2	11.4	25.0	7.8	31.3	9.1	25.8	6.4	31.9	7.7	25.8	5.5	34.8	10.6	28.2	8.3	25.6	6.1	28.6	8.4
**Positive Depression***	33.4	9.3	33.6	14.1	28.3	13.3	29.6	9.7	31.3	8.0	25.8	5.2	31.9	7.8	26.9	5.5	40.2	13.7	26.8	8.0	27.1	7.3	30.3	9.3
**Positive Panic disorder***	13.7	2.1	14.8	4.4	10.5	2.9	10.8	2.7	16.1	3.0	12.0	1.7	18.8	3.8	12.1	2.0	17.8	5.8	11.9	2.2	12.4	2.0	13.7	3.0
**Positive for all disorders**	7.1	0.9	8.3	1.6	7.2	1.8	6.0	1.8	8.7	1.2	4.6	0.4	10.5	1.3	5.5	1.3	10.8	3.0	6.6	1.1	6.1	0.8	7.3	1.5
**Positive for at least one disorder**	48.4	14.9	57.3	26.3	43.0	20.6	48.6	17.9	56.1	19.6	42.4	11.4	54.4	16.8	45.0	12.0	53.3	18.4	51.8	18.7	44.2	13.2	48.6	17.4

*(score ≥3); [1] Prevalence calculated for all respondents (N=9543); [2] Incidence calculated for respondents without any diagnosis or symptoms of mental illness before the pandemic & no experience of major stressful life event not directly related to the pandemic, compared to all respondents.

Table S5. Course of PTSD, anxiety, depression, and panic disorder

Type of mental disorder	1st assessment			1st & 2nd assessment								
	All respondents N=9543 (%)	Without preexisting mental illness & no experience of major life event N=4699 (%)	With preexisting mental illness & no experience of major life event N=1336 (%)	Without preexisting mental illness & no experience of major life event during the pandemic N=909			With preexisting mental illness & no experience of major life event during the pandemic N=301			With preexisting mental illness & experience of major stressful life event during the pandemic N=314		
				1st assessment (%) A	2nd assessment (%) A	P-value	1st assessment (%) B	2nd assessment (%) B	P-value	1st assessment (%) C	2nd assessment (%) C	P-value
**Positive PTSD**	32.4	23.2	39.7	23.8	20.2	**0.008***	37.9	33.2	0.118	53.5	49.4	0.128
**Positive GAD**	28.6	17.0	37.4	16.7	20.0	**0.016***	36.9	42.9	**0.047***	52.9	59.2	**0.043***
**Positive Depression**	30.3	19.0	41.5	17.7	20.0	0.096	41.5	42.2	0.809	54.8	53.2	0.593
**Positive Panic disorder**	13.3	6.0	21.5	5.5	6.8	0.101	21.6	20.9	0.793	35.4	35.7	0.730
**Positive for all disorders**	7.3	3.0	10.8	3.0	3.9	0.160	11.6	11.3	0.858	19.7	20.1	0.681
**Positive for at least one disorder**	48.6	35.4	63.5	35.9	33.9	0.318	63.8	62.1	0.579	76.4	76.8	0.891

* Statistically significant difference between 1st and 2nd assessment.
